# The altered expression profile of microRNAs in cardiopulmonary bypass canine models and the effects of mir-499 on myocardial ischemic reperfusion injury

**DOI:** 10.1186/1479-5876-11-154

**Published:** 2013-06-21

**Authors:** Han Qin, Guang-xian Chen, Meng-ya Liang, Jian Rong, Jian-ping Yao, Hai Liu, Zhong-kai Wu

**Affiliations:** 1 The Second Department of Cardiac surgery, The First Affiliated Hospital of Sun Yat-Sen University, 58 Zhongshan II Road, Guangzhou 510080, China; 2 Department of Anesthesiology, The First Affiliated Hospital of Sun Yat-Sen University, Guangzhou, China

**Keywords:** MicroRNA, Cardiopulmonary bypass, Ischemia reperfusion injury, Mir-499

## Abstract

**Background:**

MicroRNAs were enrolled in various cardiovascular disease especially ischemic heart diseases, but the microRNA changes during myocardial ischemia reperfusion injury underwent cardiopulmonary bypass are still unknown. This study screens the microRNA differences in CPB canines and evaluates the relationship of microRNAs with myocardial ischemia reperfusion injury.

**Methods:**

13 healthy canines received CPB with 60 minutes of aortic clamping and cardioplegic arrest, followed by 90 minutes reperfusion. Left ventricular myocardial samples, blood samples and hemodynamic data were taken at different time points. We performed microRNAs microarray experiments upon the left ventricle myocardium tissue of canines before CPB and after reperfusion for 90 minutes by pooling 3 tissue samples together and used qRT-PCR for confirmation.

**Results:**

Statistically significant difference was found in mir-499 level before CPB and after reperfusion (T1 vs. T4, p = 0.041). We further examined the mir-499 levels by using qRT-PCR in all 13 canines at 4 different time points (T1 vs. T4, p = 0.029). Mir-499 expression was negatively correlated with cardiac troponin T (cTnT) and creatine kinase- MB (CK-MB) levels of canines in all time points samples (r = 0.469, p < 0.001 and r = 0.273, p = 0.050 respectively). Moreover, higher mir-499 expression level was associated with higher dP/dt_max_ at 25 minutes and 90 minutes after reperfusion.

**Conclusion:**

Myocardial ischemic reperfusion injury with cardiopulmonary bypass results in declining level of mir-499 expression in left ventricle myocardium of canines, suggesting mir-499 would be a potential therapeutic target in cardiac protection during open heart surgery.

## Introduction

MicroRNAs are short non-coding RNAs which are about 18–25 nucleotides long and act as negative or positive regulators, primarily post-transcriptionally [1,2]. They have enrolled in various pathological conditions, especially cardiovascular disease [3], including ischemic heart disease [4], heart failure [5], cardiac hypertrophy [6] and arrhythmias [7]. Recent studies have demonstrated that there are significant expression profile changes in a bunch of microRNAs in both ischemic/normal heart tissues [8] and peripheral blood [9]. Moreover, some microRNAs perform biological functions of protecting or damaging cardiac cells in myocardial ischemia through different pathways, particularly apoptosis, which brings the treatment potency in the field of microRNAs [4,10-12].

Recently, the microRNA expression patterns in acute myocardial infarction have been fully elucidated [8,13]. However, the expression profiles of microRNAs in ischemic reperfusion (IR) injury with CPB are not yet been studied. We hypothesized that there are changes in the level of microRNAs in cardioplegic heart compared to their baseline. Those microRNAs might be potential targets for myocardial protection in CPB. To testify this hypothesis, we analyzed the expression of a broad range of microRNAs using quantitative stem-loop RT-PCR arrays on left ventricular samples at different IR time points in CPB canines, and the clinical relevance of microRNAs with myocardial biochemical markers and hemodynamic changes.

## Material and methods

### Animal care

The investigation conforms to the Guide for the Care and Use of Laboratory Animals (NIH publication no. 85–23, revised 1996). All procedures were approved by the Committee on the Ethics of Animal Experiment of the First Affiliated Hospital of Nanjing Medical University (Approval No. 2010CB5295007). All reasonable efforts were made to minimize suffering.

### Experimental protocol

**Figure 1:**
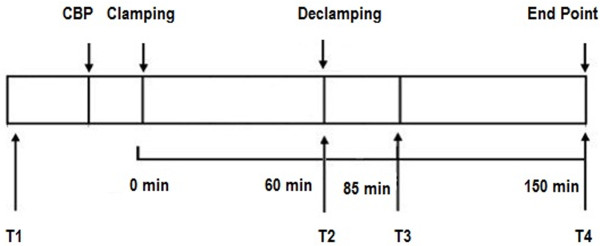
**Experimental protocol.** T1: Baseline, after thoractomy, T2: at 60 minutes after aortic clamping, T3: at 25 minutes after reperfusion, and T4: at 90 minutes after reperfusion.

Thirteen healthy adult canines (2–3 years old, weight 18.8 ± 2.16 kg, Suibei Nursery of Laboratory Animals, Baiyun District, Guangzhou) of both genders are collected. All animals accepted 60 minutes of aortic clamping and cardioplegic arrest. CPB was weaned off after reperfusion. Blood and myocardial samples from the radial artery were collected at designated time points (T1: Baseline, after thoractomy, T2: at 60 minutes after aortic clamping, T3: at 25 minutes after reperfusion, and T4: at 90 minutes after reperfusion; Figure 1).

### Anesthesia and monitoring

The canines were anesthetized with an intramuscular administration of ketamine (30 mg/kg, JiuXu Pharmaceutical Co. Ltd, Zhejiang, China), fentanyl citrate (2 μg/kg, RenFu Pharmaceutical Co. Ltd, Yi 105 Chang, China), and an intravenous administration of vecuronium bromide (0.1 mg/kg, SiDa Pharmaceutical Co. Ltd, Hainan, China) [14]. Tracheal intubation was performed, and mechanical ventilation was started at a tidal volume of 15 mL/kg body weight 110 and a rate of 15 breaths per minute using a volume cycled respirator (BIRD VELA, Model 606A, USA). The anesthesia was maintained with intravenous infusion of fentanyl, vecuronium bromide, and midazolam. The anesthesia protocol is consistent with the guidance of National Research Council Staff. Fluid-filled catheters were then placed into the left femoral artery for mean arterial pressure (MAP) monitoring and blood sampling. Fluid administration was carried out through cannulation of the left femoral vein. Blood pressure were monitored and recorded by a 16 channel chart recorder (BIOPAC MP150, California, USA).

### Cardiopulmonary bypass

CPB was performed using one roller pump for extracorporeal circulation and another roller pump for suction. We primed the extracorporeal circuit and the membrane oxygenator (AFFINITY NT 541, Medtronic Inc, USA) with canine blood (average 585 ± 76.97 mL) and multiple electrolyte injections. The total priming volume was approximately 1500 mL. The priming blood was derived from another 13 canines under general anesthesia. Heparin (XinYi Pharmacectic Manufactory of Shanghai Medicine Group, Shanghai) was added to the blood (300U/100 mL) and crystal liquid (200U/100 mL). No other medications were given to minimize any potential cross-reactions. Following preparation, heparin (300U/kg) was given intravenously for systemic anticoagulation. An 18 F arterial perfusion cannula was introduced into the aorta, while two 18 F venous cannulas (Edwards Lifesciences LLC, Irvine, USA) were placed into the right atrium and inferior vena cava, via median sternotomy. After CPB was achieved, cardiac arrest was induced by antegrade injection of homemade cold 4°C crystalloid cardioplegia (10% potassium chloride 7 mL, 25% magnesium sulfate 1.4 mL, 50% glucose 10 mL, 20% mannitol 5 mL, 5% sodium bicarbonate 18 mL, 2% lidocaine 10 mL, and 4 mg dexamethasone, with Ringer’s injection added to a total volume of 500 mL). Electric defibrillation was used if necessary after aortic declamping. Activated clotting time was maintained for more than 480 seconds. The MAP was controlled between 70 and 90 mmHg. The nasopharyngeal temperature was maintained at 36°C by a heat exchanger (Sarns Heater/Cooler, Ann Arbor, MI, USA).

### Myocardial biochemical markers determination

At T1, T2, T3, and T4, the blood sample was centrifuged and serum was extracted to measured the levels of cardiac Troponin T (cTnT) and creatine kinase- MB (CK-MB) using a high-sensitivity cardiac troponin T enzyme linked immunosorbent assay kit (USCN Life Science, Wuhan, CHN). Samples of myocardium from left ventricle were obtained using an auto-cardiac biopsy system (Bard MAGNUM REF/Cat.No. MG1522).

### Measurement of cardiac function and hemodynamic parameters

A water-filled latex balloon attached to a pressure sensor (model SP844; MEMSCAP Inc., Durham, NC) was inserted into the left ventricle through the left atrium and mitral valve. Blood pressure, maximum left ventricular developed pressure increase (dP/dt _max_) and decrease rate (dP/dt _min_) were monitored and recorded at T1, T2, T3 and T4 by a 16-channel chart recorder (BIOPAC MP150, California, 125 USA) .2.7 RNA isolation.

Total RNA was isolated from heart tissue with LCS total RNA extraction kit (TRK-1002) and with this kit genomic DNA contamination can be removed by the process of on column DNase I digestion at room temperature for 15 min, after a series of wash steps the purified total RNA was eluted with elution solution.

### Microarray analysis

For microRNAs microarray analysis, total RNA from 3 left ventricle samples of T1, and 3 samples of T4 (each T1 and T4 is paired to each canines) was isolated. After the isolation, 3 different sample of T1, each 2.5 ug total RNAs were mixed together as a pooling sample, compared with mixed 3 sample of T4. Profiling of miRNAs expression was analyzed by LC Sciences on their microarray platform using Sanger miRBase Release 17.0 database (http://microrna.sanger.ac.uk/sequences/).

The assay started from 10 μg total RNA sample, which was size fractionated using a YM-100 Microcon centrifugal filter (from Millipore) and the small RNAs (< 300 nt) isolated were 3’-extended with a poly(A) tail using poly(A) polymerase. An oligonucleotide tag was then ligated to the poly(A) tail for later fluorescent dye staining; Hybridization was performed overnight on a μParaflo microfluidic chip using a micro-circulation pump (Atactic Technologies) [15,16]. On the microfluidic chip, each detection probe consisted of a chemically modified nucleotide coding segment complementary to target microRNA (from miRBase, http://microrna.sanger.ac.uk/sequences/) or other RNA (control or customer defined sequences) and a spacer segment of polyethylene glycol to extend the coding segment away from the substrate. The detection probes were made by in situ synthesis using PGR (photogenerated reagent) chemistry. The hybridization melting temperatures were balanced by chemical modifications of the detection probes. Hybridization used 100 μL 6 × SSPE buffer (0.90 M NaCl, 60 mM Na_2_HPO_4_, 6 mM EDTA, pH 6.8) containing 25% formamide at 34°C. After hybridization detection used fluorescence labeling using tag-specific Cy5 dyes. Hybridization images were collected using a laser scanner (GenePix 4000B, Molecular Device) and digitized using Array-Pro image analysis software (Media Cybernetics). Data were analyzed by first subtracting the background and then normalizing the signals using a LOWESS filter [17] (Locally-weighted Regression). Higher signal indicates higher microRNA expression.

### Real-time PCR

10 microRNAs are selected after microarray analysis and that primers are listed in Tables 1 and 2. For Reverse transcriptase reactions it contained 250 ng total RNA, 1uM stem-loop RT primer, 5× RT buffer,10 mM each dNTPs, 40 U/ul RNase inhibitor and 200 U/ul M-MLV. Reaction mixtures were incubated in an ABI PRISM® 7900HT for 60 min at 42°C, 15 min at 70°C, and held at 4°C. Reverse transcriptase reactions including no-template controls and RT-minus controls were run in duplicate. Real-time PCR was performed using Platinum SYBR Green qPCR SuperMix-UDG (Invitrogen). The reaction mixtures were incubated in a 96-well plate at 95°C for 30 sec, followed by 40 cycles of 95°C for 5 sec and 60°C for 30 sec. All reactions were run in triplicate. To account for possible differences in the amount of starting RNA, microRNA expressions were normalized to small RNU6B, with the similar efficiency of microRNAs [18,19]. Data of qRT-PCR were demonstrated by nominal CT value (normalized to RNU6B), and fold changes were calculated by 2^|△△CT|. Higher nominal CT value means lower microRNA expression level.

**Table 1:** Primers for reverse transcription of microRNAs

**No.**	**Assay name**	**Gene Bank/miRbase Access No.**	**Primers for reverse transcriptase Sequence(5' to 3')**
1	cfa-miR-451	MIMAT0009870	AAACCGUUACCAUUACUGAGUU
2	cfa-miR-423	MIMAT0006742	UGAGGGGCAGAGAGCGAGACUUU
3	cfa-miR-23	MIMAT0006640	AUCACAUUGCCAGGGAUUU
4	cfa-miR-133	MIMAT0009835	UUUGGUCCCCUUCAACCAGCUA
5	cfa-miR-489	MIMAT0009860	GUGACAUCACAUAUACGGCGGC
6	cfa-miR-574	MIMAT0006673	CACGCUCAUGCACACACCCACA
7	cfa-miR-214	MIMAT0009847	ACAGCAGGCACAGACAGGCAGU
8	cfa-miR-1	MIMAT0006656	UGGAAUGUAAAGAAGUAUGUA
9	cfa-miR-494	MIMAT0009905	UGAAACAUACACGGGAAACCUC
10	cfa-miR-499	MIMAT0006655	UUAAGACUUGCAGUGAUGUUU
Control	human-RNU6B	NR_002752	

**Table 2:** Primers for real time PCR

**Assay name**	**Forward Primer(5' to 3')**	**Reversed Primer(5' to 3')**
cfa-miR-451	AGCCGGTAAACCGTTACCAT	CAGTGCAGGGTCCGAGGTAT
cfa-miR-423	CTGTTGTGAGGGGCAGAGAG	CAGTGCAGGGTCCGAGGTAT
cfa-miR-23	GGACGTCATCACATTGCCAG	CAGTGCAGGGTCCGAGGTAT
cfa-miR-133	GGTGTTTGGTCCCCTTCAAC	CATAAGCCAGCGCGATCAG
cfa-miR-489	GGCGCTGGTGACATCACAT	CAGTGCAGGGTCCGAGGTAT
cfa-miR-574	TGTCACGCTCATGCACACA	CAGTGCAGGGTCCGAGGTAT
cfa-miR-214	TTGGACAGCAGGCACAGACA	CAGTGCAGGGTCCGAGGTAT
cfa-miR-1	TTGCGCTGGAATGTAAAGAAG	CAGTGCAGGGTCCGAGGTAT
cfa-miR-494	CGGGTGAAACATACACGGGA	CATAAGCCAGCGCGATCAG
cfa-miR-499	AGGCCGGTTAAGACTTGCAGT	CATAAGCCAGCGCGATCAG
human-RNU6B	CAAATTCGTGAAGCGTTCCATA	AGTGCAGGGTCCGAGGTATTC

### Statistical analysis

All values are presented as mean ± standard deviation. Results of cardiac enzymes, hemodynamic parameter changes, microarray and qRT-PCR results between time intervals were analyzed by paired t test. Spearman correlation coefficients were used to examine the relationship between miR-499 and cardiac enzymes levels. P < 0.05 was considered statistically significant.

## Results

### Myocardial biochemical markers changes during cardiopulmonary bypass and reperfusion

**Figure 2:**
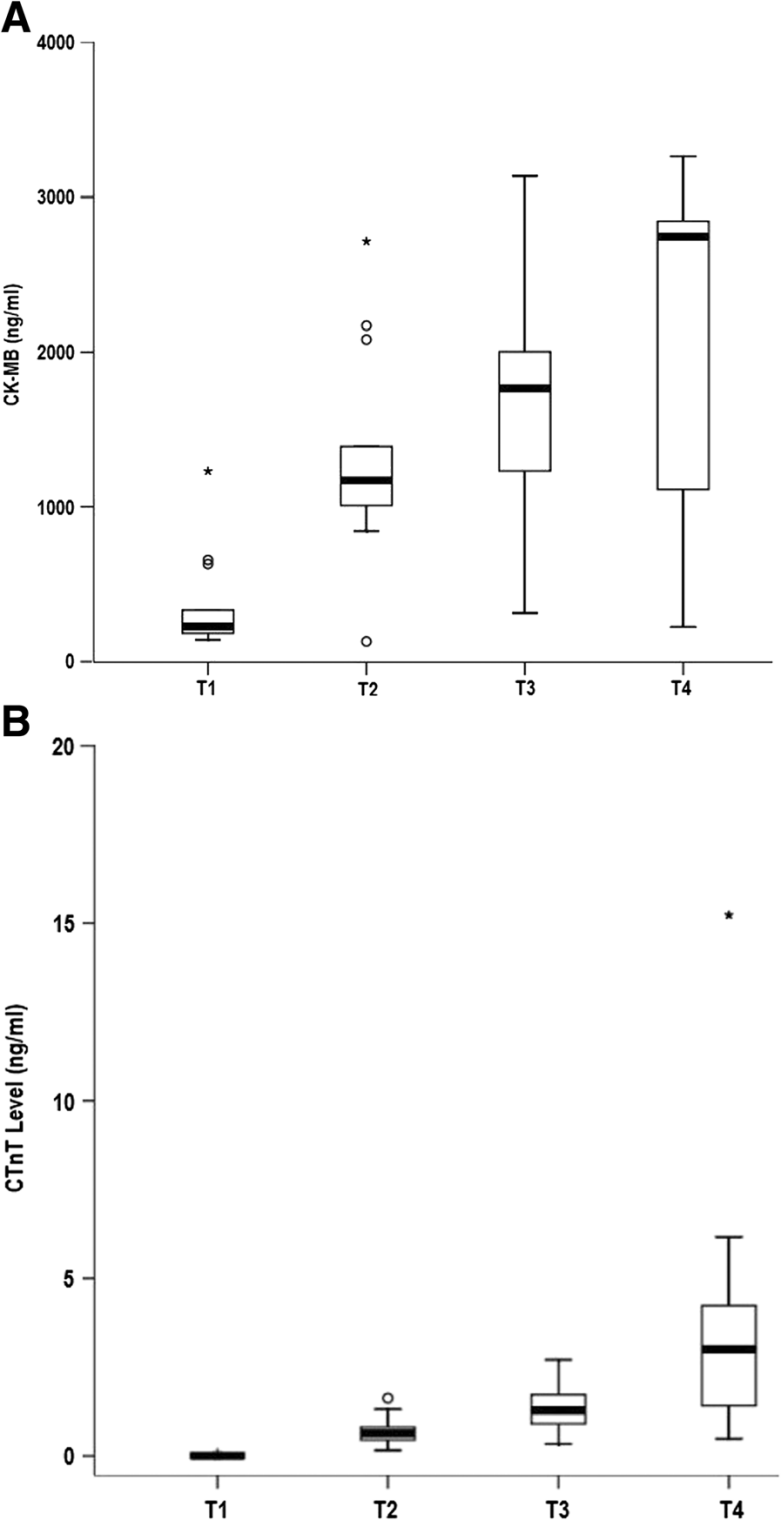
**(A and B) The levels of blood CK-MB and cTnT are significantly increased after CPB and reperfusion.** P <0.001 were found between T1 vs.T2, T1vs.T3, T1vs.T4.

As demonstrated in Figure 2A and B, the level of cTnT and CK-MB level in blood was raised during ischemia and reperfusion. The level of cTnT in T1, T2, T3, T4 were 0.015 ± 0.020, 0.707 ± 0.392, 1.356 ± 0.694, 3.771 ± 3.864 ng/ml respectively and the level of T2, T3 and T4 were significantly higher than T1 respectively (T1 vs. T2, p < 0.001; T1 vs. T3, p < 0.001; T1 vs. T4, p < 0.001). The level of CK-MB in T1, T2, T3, T4 were 357.46 ± 311.85, 1311.92 ± 667.58, 1667.31 ± 808.39, 2165.46 ± 1050.63 ng/ml and the level of T2, T3 and T4 were significantly higher than T1 respectively (T1 vs. T2, p < 0.001; T1 vs. T3, p < 0.001; T1 vs. T4, p < 0.001).

### Microarray results

Microarray data mining and differential analyses of expression of microRNAs has found 19 significantly changed microRNAs associated with ischemia and reperfusion in CPB animals in the left ventricular myocardium with CPB and reperfusion (T1/T4) (Table 3). Mir-451, mir-1, mir-489, mir-133b/c, mir-143, mir-30b/c, mir-191, mir-22 are down regulated, whereas mir-423a, mir-494, mir-214, mir-574, mir-23a/b, let-7e, mir-499, mir-24 are up regulated.

**Table 3:** MicroRNAs differentially expressed in IR myocardium of canines with CPB and reperfusion by Microarray

**microRNA**	**Control (T1)/reperfusion (T4)(signals)**	**Fold change**	**P value**
cfa-miR-451	2239/548	0.24	1.07E-14
cfa-miR-1	10512/1379	0.13	1.09E-13
cfa-miR-423a	440/768	1.74	1.38E-10
cfa-miR-494	271/1914	2.82	2.80E-10
cfa-miR-489	791/386	7.06	2.05E-09
cfa-miR-214	382/632	1.65	2.37E-09
cfa-miR-574	318/1020	3.20	4.55E-09
cfa-miR-133c	3782/2612	0.69	1.19E-08
cfa-miR-143	1223/853	0.69	4.91E-08
cfa-miR-133b	3502/2515	0.71	3.64E-07
cfa-miR-23a	914/1467	1.60	3.94E-07
cfa-miR-30c	1436/823	0.57	1.40E-06
cfa-let-7e	518/981	1.89	2.90E-06
cfa-miR-23b	663/1197	1.80	5.52E-06
cfa-miR-499	1012/1296	1.28	8.70E-04
cfa-miR-191	964/831	0.86	1.41E-03
cfa-miR-30b	756/572	0.75	1.63E-03
cfa-miR-22	1732/1493	0.86	1.77E-03
cfa-miR-24	3381/3759	1.11	2.71E-03

Microarray analysis showed that totally 19 microRNAs were altered, in which 10 microRNAs were down-regulated and 9 microRNAs were up-regulated.

### Confirmation of the altered expression of microRNAs by qRT-PCR

To verify the accuracy of the microarray results above, we selected 10 of the 19 deregulated microRNAs according to relatively lower P value in microarray or its association to myocardial infarction by various researches. For further confirmation, qRT-PCR test was used in 3 pair samples. Only changes in cfa-mir-499 between T1 and T4 had reached statistical significance (p = 0.041) (Table 4).

**Table 4:** microRNA differentially expressed in control and after CPB/ reperfusion

**microRNA**	**Fold Change(T4/T1 )**	**P value**
Mir-499	0.36	0.041
Mir-423	0.60	0.077
Mir-1	0.33	0.098
Mir-23	0.38	0.171
Mir-574	0.55	0.176
Mir-494	1.69	0.231
Mir-133	0.83	0.242
Mir-214	0.90	0.737
Mir-489	0.97	0.927

Mir-499 was found down-regulated at 90 minutes after reperfusion (T4) compared with Baseline (T1) in 3 pair samples by qRT-PCR. Significant change was found in mir-499 expression level (P < 0.05).

### Mir-499 expression among different time points

**Figure 3:**
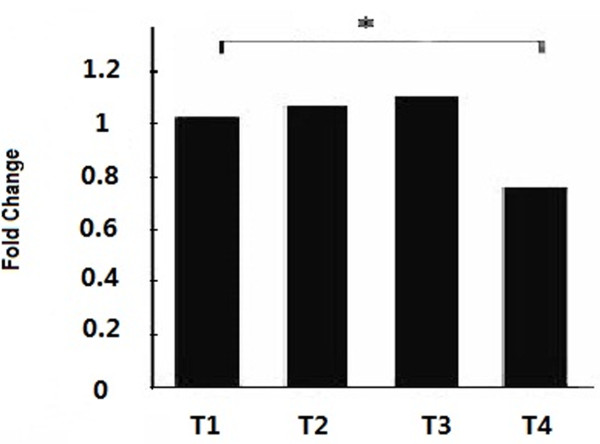
**A comparison of fold changes based on qRT-PCR analysis of mir-499 of 4 time points.** (T1 vs. T4). Mir-499 was down-regulated after 90 minutes reperfusion (T4) compared with baseline (T1). (*P = 0.029).

Mir-499 expression was examined in 4 different time points in all 13 canines. As shown in Figure 3, the mir-499 expression levels in T4 (at 90 minutes after reperfusion) were significantly lower than those in T1 (p = 0.029). There’re no statistical differences between T1and T2 (p = 0.821), so does T1 and T3 (p = 0.577).

### Correlation of mir-499 expression and cTnT levels at all time points

**Figure 4:**
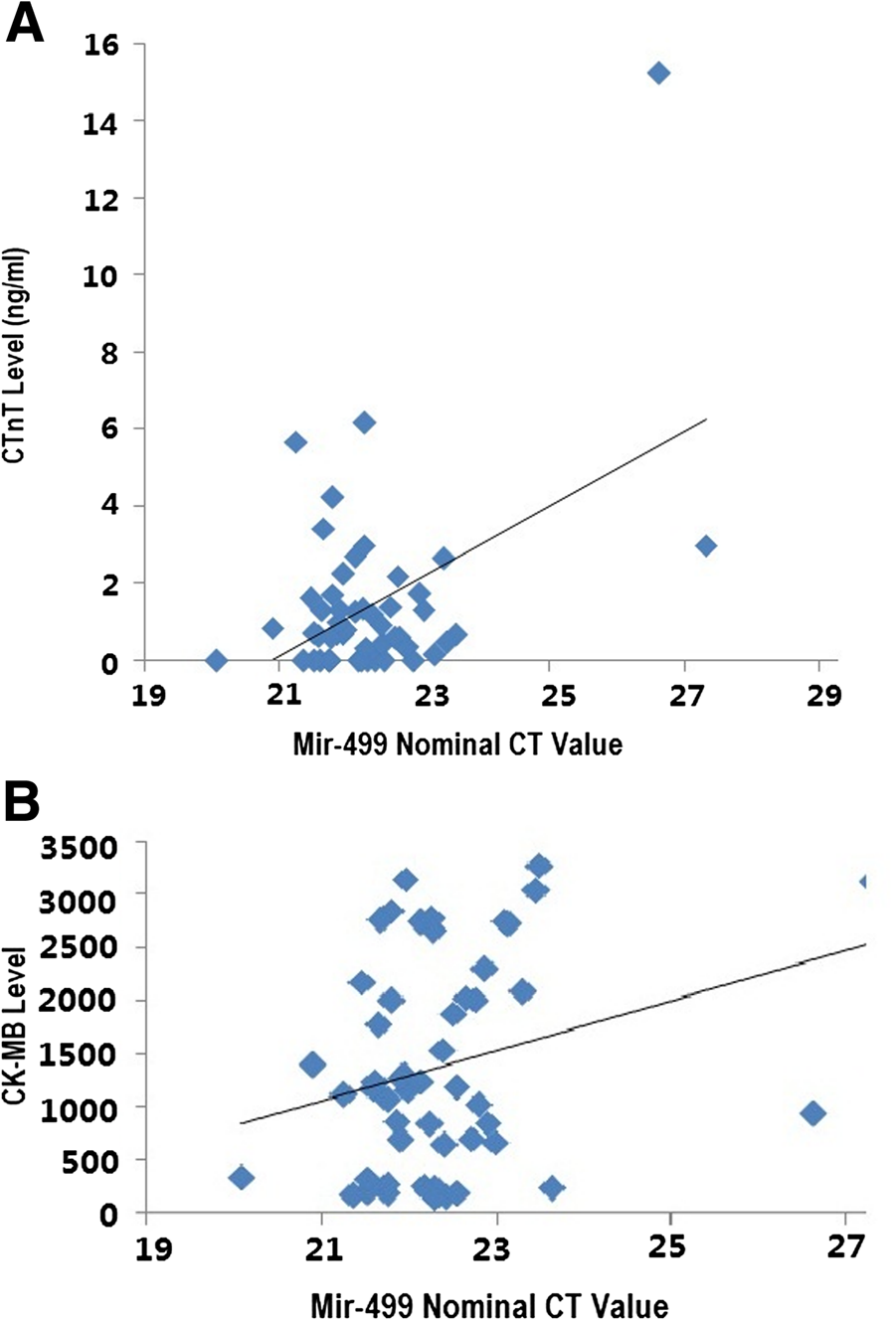
**(A and B) Correlation plots between cTnT, CK-MB levels and mir-499 nominal CT values in all 52 samples (all 4 time points).** Higher CT value means lower mir-499 expression. The higher the cardiac enzymes, the higher the nominal CT value of mir-499 thus the lower the mir-499 expression level. (**A**) mir-499 vs. cTnT (r = 0.469, p < 0.001). (**B**) mir-499 vs. CK-MB (r = 0.273, p = 0.050).

Because of high degree of dispersion, we use nominal CT value instead of fold changes in correlation analysis (Figure 4A and B). Nominal CT value of mir-499 were positively correlated with cTnT and CK-MB levels at all time points (r^2^ = 0.469, p < 0.001 and r^2^ = 0.273, p = 0.050 respectively), indicating that the higher the cTnT and CK-MB in blood, the lower the mir-499 expression level in myocardium.

### Hemodynamic parameters and mir-499 expression level

A cut-off value was set in the median level of mir-499 expression in T3 by nominal CT value and divided 13 canines into 2 groups. The hemodynamic parameters between groups were compared using paired t test. As shown in Table 5, higher mir-499 expression level in T3 is associated with higher dP/dt_max_ (maximum left ventricular developed pressure increase rate) in T3 and higher dP/dt_max_ in T4.

**Table 5:** Association between mir-499 level in T3 and hemodynamic parameters

	**T3**	**p-value**	**T4**	**p-value**
Mean dP/dt_max_ with higher mir-499 expression (mmHg)	1107.86 ± 318.10	0.009	1116.00 ± 310.10	0.019
Mean dP/dt_max_ with lower mir-499 expression (mHg)	620.67 ± 215.58		629.33 ± 326.55	
Mean dP/dt_min_ with higher mir-499 expression (mmHg)	605.57 ± 369.99	0.209	645.29 ± 328.58	0.076
Mean dP/dt_min_ with lower mir-499 expression (mmHg)	360.60 ± 273.99		321.00 ± 256.24	

Higher mir-499 expression level in T3 is associated with higher myocardial systolic function in T3 and T4. dP/dt _max_ = maximum left ventricular developed pressure increase rate; dP/dt _min_ = maximum left ventricular developed pressure decrease rate.

## Discussion

Our study provides evidence that several microRNAs’ level change during myocardial IR injury in CPB canines, which are found in left ventricular myocardiums using microRNA array with pooling samples. Mir-1, mir-494, mir-133, mir-499, mir-214 have been reported to be associated with acute myocardial infarction [8,11,13,20-22]. Some of the altered microRNAs were reported to be related to apoptosis (mir-191 [23], mir-22 [24], mir-30 [25], mir-24 [26]), hypoxia(mir-451 [27], mir-23 [28]) and cell proliferation(mir-489 [29], let-7e [30]). These results indicate that a bunch of microRNAs may take part in response to myocardial IR injury. Among aberrantly expressed microRNAs in microarray which were verified by qRT-PCR, mir-499 was found to be a significantly down-regulated microRNA at 90 minutes following reperfusion exclusively. However, the qRT-PCR shows ambivalent results in mir-499 expression compared with results given by microarray. Possible reasons included the disturbance of pooling sample, and the relative inaccuracy of microarray. The present study is the first report to address cardiac microRNAs in a clinical relevant CPB model. Furthermore, there is a moderate negative correlation between mir-499 level, cardiac enzymes levels and hemodynamic parameters, which supports previous studies that mir-499 is in association with cardiomyocytes damage and prognosis. Therefore, our results imply that mir-499 might possibly be a potential therapeutic target of myocardial protection during CPB surgery.

Recently, it was reported that myocardial infarction results in great changes of microRNAs level in myocardium and peripheral blood among patients and animal models [8,9,21]. The molecular mechanisms regulating microRNA expression in cardiac ischemia and reperfusion remain largely unknown [31]. However, such pathophysiological conditions closely related to cardiomyocyte apoptosis through which specific microRNAs took roles in preventing and maintaining an effect of cardio-protection [32]. For instance, mir-494 is down regulated in ischaemic hearts and has a cardioprotective effects by activating the Akt pathway [20]. Mir-133 produces opposing effects on apoptosis by targeting heat shock protein 60/70 and caspase-9 in cardiomyocytes [33]. Notwithstanding, most of current studies are base on the model of index heart ischemia mimicking acute coronary syndrome, but the changes in cardiopulmonary bypass still remain uncertain. Recent findings suggest that serum and urine mir-1 act as biomarker of myocardial injury with cardiopulmonary bypass [34]. CPB can trigger apoptosis during cardiac surgery by a series of mechanisms, including ischemia, oxidative stress upon reperfusion, contact with CPB circuit material, proinflammatory cytokines, etc. [35,36]. Most of the myocardial IR injury in CPB are stunning while myocardial infarction are more common in acute coronary syndrome [37]. Malmberg discovered that short and unprotected warm ischemia produces much more cardiomyocytes apoptosis than a longer period of protected cold ischemia [35]. Thus, current articles about microRNA results in myocardial ischemia can’t be simply utilized in myocardial IR injury of CPB models. Our results demonstrated that among several microRNAs which were found fluctuating following IR injury, only mir-499 was down-regulated. Mir-1, mir-494, mir-133, which shown significant changes in index ischemia [13,20], can’t demonstrate significant changes during CPB.

Mir-499 is a cardiac ventricular abundant microRNA under physiological condition and evolutionarily conserved among species [38-42]. It is encoded by β-myosin heavy chain gene (*Myh7b*) [43,44] and shown a pivotal role in inhibiting cardiomyocytes apoptosis. Researchers noted that mir-499 was down regulated in myocardial ischemia in rats [22] and correlated to the severity of cardiomyocytes damage [45]. However, our results show that mir-499 fail to decrease after clamping the aorta for 60 minutes. Such phenomenon might origin from the protection effect of hypothermia and cardiaplegic arrest. At 90 minutes after reperfusion, the mir-499 level decrease significantly compared with baseline. This is the first time to demonstrate that mir-499 related to CPB myocardial reperfusion injury, rather than ischemia injury. Furthermore, Mir-499 targets alpha, beta isoforms of the calcineurin catalytic subunit and dynamin-related protein-1 (Drp1) in mitochondria during myocardial infarction [22], suggesting that mitochondrial activation pathway takes an important role in cardiomyocytic apoptosis after reperfusion in CPB, which is accordance with previous reports [46].

In the present study, we observed that lower myocardium level of mir-499 was associated with poorer cardiac systolic function during reperfusion than their counterparts. What’s more, mir-499 level in myocardium negatively correlated to cTnT level of peripheral blood, giving a clue that myocardial level of mir-499 might reflect the degree of cardiomyocyte damage. Elevated level of mir-499 in blood of coronary sinus was discovered from patients suffering from troponin-positive acute coronary syndromes and significantly correlated to hsTnT [45]. Moreover, plasma mir-499 was identified as a biomarker and prognostic factor in acute myocardial infarction [47,48]. Our results confirm and extend the concept of mir-499 in previous reports, showing that the decrease of mir-499 in myocardium might be associated with myocardial injury with a significant release of microRNAs into peripheral circulation.

## Conclusions

In principle, the present findings support the hypothesis that microRNAs expression level can be influenced by IR injury in CPB models and IR injury resultes in a decreased level of mir-499 during reperfusion. Lower mir-499 level correlated to more severe IR injury and poor hemodynamic performance. However, the mechanism between microRNAs and IR injury in CPB are not fully understood. Whether the decreased expression level of mir-499 after reperfusion is the cause or the consequence of myocardial injury is still uncertain. We used pooling sample which may diminished the differences for microRNAs array and decrease the sensitivity to examine the unknown microRNAs which participated in IR injury mechanism. Our present study suggested that the introduction of mir-499 as a potential therapeutic method of myocardial protection in open heart surgery would be possible but still wait for further studies.

## Competing interests

The authors declare that they have no competing interests.

## Authors’ contributions

HQ carried out the molecular studies, participated in the sequence alignment and drafted the manuscript. GXC, MYL, JPY, HL participated in establishing cardiopulmonary bypass, Jian Rong participated in dog anaesthesia. HQ, ZKW participated in the design of the study and performed the statistical analysis. ZKW, GXC, MYL conceived of the study, and participated in its design and coordination and helped to draft the manuscript. All authors read and approved the final manuscript.
